# Insight on Efficacy of Renal Artery Denervation for Refractory Hypertension with Chronic Kidney Diseases: A Long-Term Follow-Up of 24-Hour Ambulatory Blood Pressure

**DOI:** 10.1155/2022/6895993

**Published:** 2022-09-21

**Authors:** Han Cai, Zhoufei Fang, Ruofan Lin, Wenqin Cai, Ying Han, Jinzi Su

**Affiliations:** ^1^Department of Cardiology, The First Affiliated Hospital of Fujian Medical University, Fuzhou, China; ^2^Fujian Hypertension Research Institute, The First Affiliated Hospital of Fujian Medical University, Fuzhou, China; ^3^Department of Geriatrics, The First Affiliated Hospital of Fujian Medical University, Fuzhou, China; ^4^Clinical Research Center for Geriatric Hypertension Disease of Fujian Province, The First Affiliated Hospital of Fujian Medical University, Fuzhou, China; ^5^Branch of National Clinical Research Center for Aging and Medicine, The First Affiliated Hospital of Fujian Medical University, Fuzhou, Fujian, China; ^6^The First Clinical Medical College of Fujian Medical University, Fuzhou, China

## Abstract

**Aims:**

To explore the long-term efficacy and safety of renal denervation in patients with RHT and CKD, a post hoc analysis of eGFR subgroups was completed.

**Methods:**

Fifty-four patients with refractory hypertension with chronic kidney disease were treated with RDN and enrolled in the study. Patients were divided into three groups according to eGFR: eGFR 46–90 ml/min group, eGFR 15–45 ml/min group, and eGFR <15 ml/min group. The planned follow-up period was 48 months to assess 24 h ambulatory blood pressure, renal function, type of antihypertensive medication, and RDN complications.

**Results:**

The ablation sites of the GFR 46–90 ml/min group and GFR 15–45 ml/min group were 32.57 ± 2.99 and 29.53 ± 5.47, respectively. No complications occurred in the GFR 46–90 ml/min group. The GFR<15 ml/min group was treated with 27.07 ± 5.59  ablation. Renal artery dissection occurred in each group of GFR 15–45 ml/min and GFR <15 ml/min. And renal stent implantation artery was performed on these two patients. No severe renal artery stenosis occurred. There were no significant differences in Scr and eGFR between the three groups at each follow-up point. Compared with baseline, SBP was significantly of each group decreased to varying degrees at each follow-up time point. SBP decreased most in the GFR 46–90 ml/min group. Compared with baseline, the type of antihypertensive drugs used in the GFR46-90 ml/min group decreased significantly except for 36 and 48 months. At 48 months' postadmission, there was a significant decrease in the type of antihypertensive medication used in the GFR15-45 ml/min group, and there was no significant decrease in the type of antihypertensive medication used in the GFR<15 ml/min group.

**Conclusions:**

RDN can safely reduce SBP in CKD patients combined with RHT for 48 months, with the most pronounced reduction in the GFR15-45 ml/min group. The variety of antihypertensive drugs was significantly reduced after RDN. This was particularly evident in patients with GFR 15–45 ml/min.

## 1. Introduction

Chronic kidney disease (CKD) is a common clinical disease with a global prevalence of 8–16% and 10.8% in China [1, 2]. 40.4% of CKD patients with hypertension are refractory hypertension (RHT) [3]. Compared with normal hypertensive patients, RHT patients with CKD have more uncontrollable blood pressure and higher risk of cardiovascular events [4]. Uncontrollable blood pressure promotes the development of chronic kidney disease and eventually leads to end-stage renal disease (ESRD). ESRD is characterized by a series of clinical manifestations caused by water, electrolyte, acid-base balance disorder, renal endocrine dysfunction, and accumulation of endogenous toxic substances and is the most serious stage of chronic renal failure. The incidence of RHT is higher [5]. RHT is defined as the failure to control blood pressure within the normal range (140/90 mm·Hg) after 4 weeks despite adequate combination of more than three antihypertensive drugs, including diuretics.

RHT is known to be a major risk of cardio-cerebral vascular events and poor prognosis of CKD patients [6, 7]. The mechanism of CKD complicated with RHT is complex. Several previous studies revealed that activation of renin angiotensin system (RAS) and sympathetic hyperexcitability play a predominant role in the development of RHT in patients with ESRD. The water and sodium retention and removal of antihypertensive drugs by dialysis may be also involved [8]. Previous evidence has revealed that the changes in the dialysis methods, such as increased peritoneal dialysis, high-throughput dialysis, low-sodium dialysis, and optimized antihypertensive drugs, such as aldosterone receptor antagonists combined with angiotensin-converting enzyme inhibitors and *β*-blockers, could control blood pressure in some CKD patients combined with RHT [9, 10].

In recent years, the interventional therapy such as renal artery embolization, nephrectomy, and renal artery stent have been noted as better treatments for some special types of CKD patients, including those complicated with massive proteinuria, renal tumor, or severe renal artery stenosis [11–13]. Renal artery denervation (RDN) is a newly proposed interventional therapy, which could lower blood pressure by improvement of central hypertension set point caused by injuring renal sympathetic nerve fibers and inhibiting sympathetic hyperexcitability. RDN was successful in Symplicity HTN-1 trial and Symplicity HTN-2 trial, but the negative results of Symplicity HTN-3 trial have been widely controversial. Recently, some clinical trials, such as the Global Symplicity Study, Spyral HTN off-med trial, Denger-HTN, have demonstrated that not all RHT patients are suitable for RDN and that it may be effective for patients with sympathetic hyperexcitability. Unfortunately, there is no accurate method to evaluate the sympathetic excitability in clinical practice currently [14]. The operating mode of RDN has a significant impact on the results. Combined ablation of trunk and branches, increased ablation points, and increased total ablation energy and time may be more effective [11]. In the previous RDN related studies, patients with severe CKD such as ESRD were mostly used as the exclusion criteria, or only a few patients were included. During this period, there has also been a growing recognition of limitations of the definition and classification, leading to a heated debate and calls for revisions. Such debates reflect critical self-appraisal of changing knowledge and practice within the discipline and provides opportunities for improvement.

**Figure 1:**
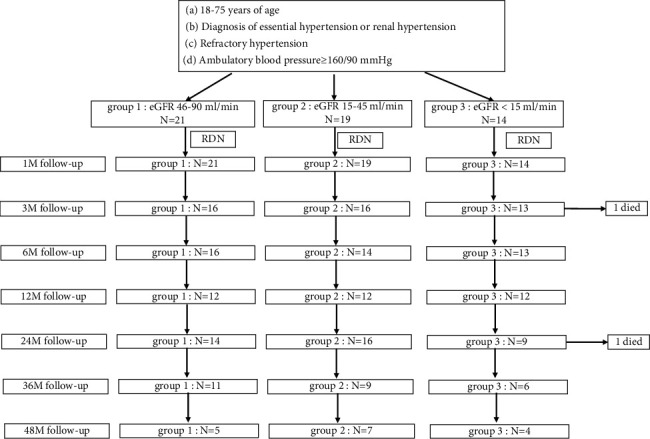
Flowchart of the study.

To date, the definition and classification of chronic kidney disease was proposed by the National Kidney Foundation Kidney Disease Outcomes Quality Initiative (NKF-KDOQI) in 2002 [15] and endorsed by the Kidney Disease: Improving Global Outcomes (KDIGO) in 2004 [16]. It is worth noting that CKD3 was divided into 3a and 3b stages in 2012 revision [17]. By comparing the clinical outcomes of stage 3b and stage 3a, it was found that 3b stage had high mortality and poor prognosis. Therefore, the precise period of CKD combined with RHT intervention directly affected the clinical outcome. This study focused on patients combined with CKD and RHT. According to different GFR stages, the changes in blood pressure, renal function, drug types, and clinical prognosis of each group before and after RDN intervention were compared. This was a prospective cohort study in which CKD patients with RHT were treated with RDN after achieving positive results, and we further explored the optimal intervention period (Figure 1).

## 2. Methods

### 2.1. Study Design

Fifty-four patients diagnosed with CKD combined with RHT and performed by RDN operation from January 2016 to January 2021 in The First Affiliated Hospital of Fujian Medical University were approached for enrollment in this prospective study. Patients were divided into three groups according to eGFR: Group1 (eGFR46-90 ml/min), Group2 (eGFR15-45 ml/min), and Group3 (eGFR<15 ml/min). The eGFR calculation method based on KDIGO 2021 clinical practice guideline for the management of glomerular diseases [18]. The primary objective is to assess procedural and long-term safety and efficacy of RDN in a real-world setting, with recommended follow-up for 48 months. A contact photoinduced pressure unipolar ablation catheter was delivered to the aorta and bilateral renal arteries, and a three-dimensional model was established using ENSITE for point-by-point ablation in a four-quadrant spiral from far-to-near. The intraoperative parameters and catheter operation methods of patients were recorded.

This study is a registration study, which has been approved by the Ethics Committee of The First Affiliated Hospital of Fujian Medical University. This is a cohort study which was successfully registered in the Chinese Clinical Trial Registry, under registration number: ChiCTR-ONC-17012483.

The numbers for each follow-up are those of patients who attended each predefined visit at the time. Two patients died and lost to follow-up at the visit time. Data from patients with at least 48 months of follow-up were included in the final analysis (*n* = 54).

### 2.2. Inclusion Criteria

Patients were eligible for RDN only if (a) they had a diagnosis of essential hypertension or renal hypertension between the ages of 18 and 75 years, (b) RHT criteria were prescribed with an adequate combination of 3 or more antihypertensive drugs for over 4 weeks, usually including a long-acting calcium channel blocker, a blocker of the renin-angiotensin system (angiotensin-converting enzyme inhibitor, or angiotensin receptor blocker), and a diuretic, (c) they had a blood pressure ≥160/90 mm·Hg in the clinic 3 times [19], and (c) hypertensive patients were willing to participate in this clinical trial and signed informed consent and then hospitalized for further evaluation.

### 2.3. Exclusion Criteria

Exclusion criteria were as follows: (a) those with unilateral kidney, renal artery stenosis of more than 50%, renal tumor, history of renal surgery, or interventional surgery; (b) pregnant or lactating women; (c) those with previous history of arteriosclerotic cerebrovascular disease (ASCVD) and loss of self-care ability; (d) those with severe systemic infection; (e) 24-hour ambulatory blood pressure suggests systolic blood pressure <135 mm·Hg; and (f) those with secondary hypertension.

### 2.4. Clinical Data

**Figure 2:**
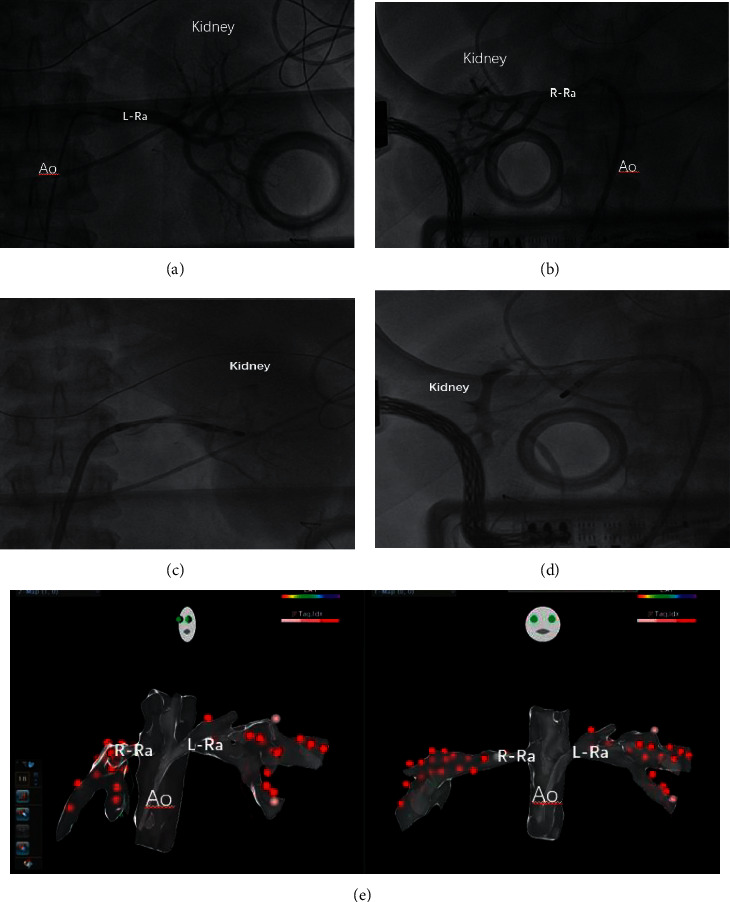
RDN operation. (a) Left renal arteriography; (b) right renal arteriography; (c, d) contact photoinduced pressure unipolar ablation catheter was delivered to the aorta and bilateral renal arteries; (e) three-dimensional map anatomy was established using the ensite system for point-by-point ablation in a four-quadrant spiral. The left picture shows the left-anterior oblique view, and the right one shows a-p position. L = left, R= right, RA = renal artery, and AO = aorta.

(a) We collect the clinical data of patients at baseline and follow-up for 1 week, 1 month, 3 months, 6 months, 12 months, 24 months, 36 months, and 48 months, including age, gender, height, weight, heart rate, and duration of hypertension. Systolic blood pressure (SBP) and diastolic blood pressure (DBP) were collected by 24 h blood pressure (Switzerland schiller MT300). (b) Hematological indicators included serum creatinine (Scr), estimated glomerular filtration rate hemoglobin (eGFR), thrombocyte, glycosylated hemoglobin, total bilirubin, albumin, potassium, sodium, uric acid, total cholesterol, triglyceride, low-density lipoprotein cholesterol (LDL), and high-density lipoprotein cholesterol (HDL). (c) Echocardiographic (Vivid E9) indicators consisted of left atrial diameter (LAD), left ventricular end diastolic diameter (LVEDD), interventricular septal thickness (IVST), left ventricular ejection fraction (LVEF), and ratio of early diastolic mitral flow velocity to early diastolic mitral annulus tissue velocity (E/*e*) (Figure 2).

### 2.5. RDN Procedure

(a) Preoperative preparation: after anesthesia with 1% lidocaine, the right femoral artery was punctured with the modified Seldinger technique, and an 8F artery sheath was indwelling (Cordis, USA), and 100 U/kg heparin was injected into the sheath; (b) renal angiography: a 6F JR3.5 angiography catheter (Cordis, USA) or a 5F MPA multifunctional catheter (Cordis, USA) was inserted into the trunk of the left and right renal arteries; (c) RDN methods: a 8F RDC catheter (Cordis, USA) was sent into the proximal of the left and right renal arteries, a contact photoinduced pressure unipolar ablation catheter (Abbott, USA) was delivered to the aorta and bilateral renal arteries, and a three-dimensional model was established using ENSITE for point-by-point ablation in a four-quadrant spiral from far-to-near. The ablation power of the trunk of renal artery was 12 W, and the branch was 8–10 W. Ablation time was 40 seconds per point; (d) renal arteriography was performed after RDN to assess whether complications occurred [20].

### 2.6. Complications of RDN

Some complications of RDN include the following: (a) severe renal artery stenosis over 50% of vascular diameter; (b) the renal artery dissection affects blood flow; (c) the femoral artery pseudoaneurysm or arteriovenous fistula; (d) death.

### 2.7. Statistical Analysis

Data were analyzed with SPSS 23.0 (SPSS Inc., Chicago, IL, USA). Normal distribution count data were expressed as mean ± standard deviation. Categorical variables were presented as percentages. Comparison within the group was done using paired *t-*test. Comparison between the groups was done using the SNK test. Repeated measures ANOVA was used before and after treatment (baseline, 1 wks, 1 mth, 3 mths, 6 mths, 12 mths, 24 mths, 36 mths, and 48 mths), and Bonferroni correction was used for further pairwise comparisons. The chi-square test was used to compare the statistical data between the groups. Nonnormal distribution measurement data were expressed as P50 (P25, P75). The nonparametric test was applied to the differences between the nonnormal data groups. A two-sided *P* value less than 0.05 was considered significant (Table 1).

## 3. Results

### 3.1. Characteristics of Study Patients

As shown in Table 1, a total of 54 patients were included in the present study. Twenty-one patients enrolled in the GFR46-90 ml/min group. Scr was (1.10 ± 0.22) mg/dl. Nineteen patients enrolled in the GFR 15–45 ml/min group. Scr was (2.72 ± 0.88) mg/dl. Fourteen patients enrolled in the GFR <15 ml/min group. Scr was (9.71 ± 4.37) mg/dl.

Mean body mass index, hemoglobin, albumin, and potassium at baseline were statistically different among the groups (all *P* < 0.010).

### 3.2. Efficacy and Safety Assessments during RDN (Table 1)

Safety outcomes are shown in Table 1. During the RDN procedure, ablation sites showed 32.57 ± 2.99 in the GFR 46–90 ml/min group and 29.53 ± 5.47 in the GFR 15–45 ml/min group. No complications occurred in the GFR 46–90 ml/min group. 27.07 ± 5.59 ablation treatments were applied in GFR <15 ml/min group. Renal artery dissection occurred in each group of GFR 15–45 ml/min and GFR <15 ml/min. And renal stent implantation artery was performed on these two patients. No severe renal artery stenosis over 50% of vascular diameter happened. One patient died at 3- and 12-month follow-up in the GFR 15–45 ml/min group. Direct causes of death were underlying diseases, irrelevant to RDN operation.

### 3.3. Changes of 24 h Ambulatory Blood Pressure and Antihypertensive Drugs

**Figure 3:**
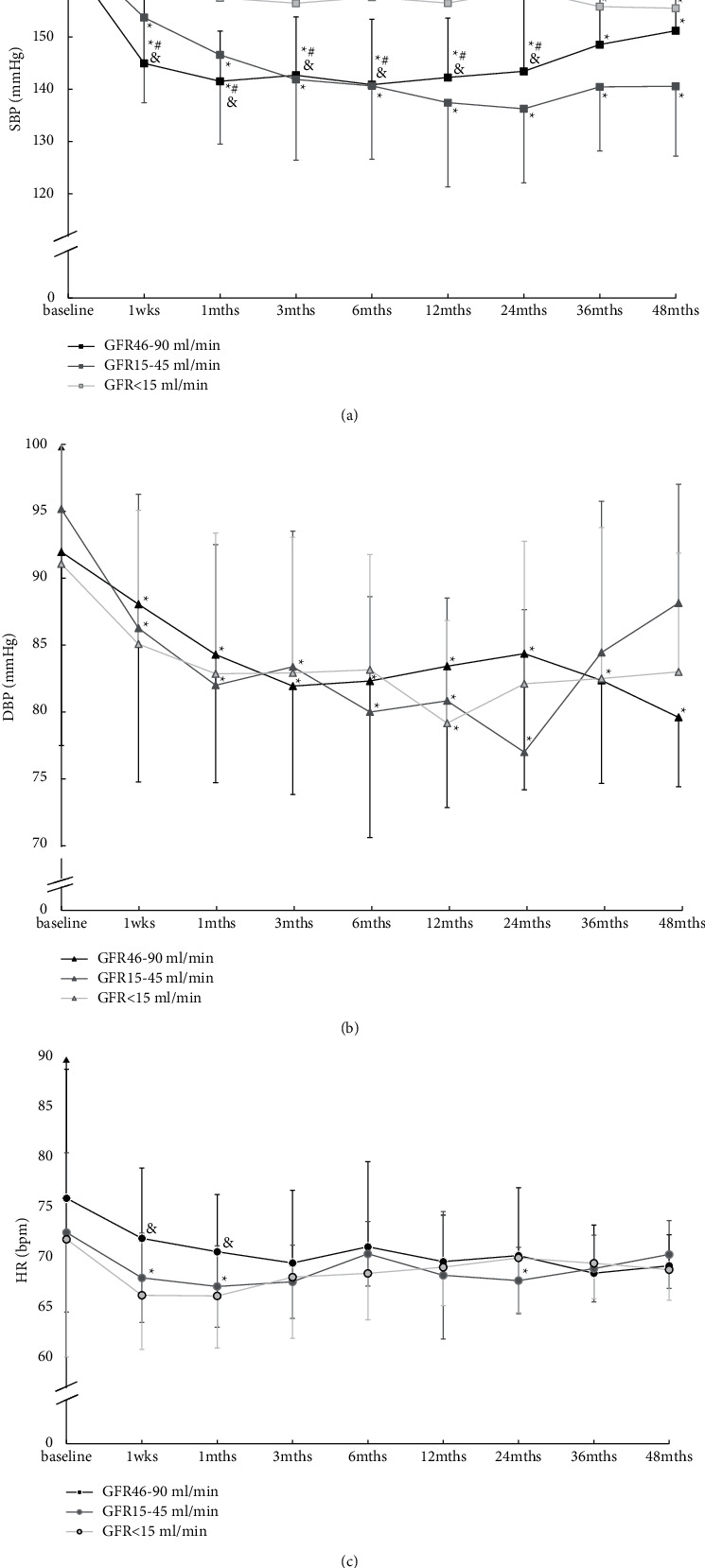
Changes of 24 h ambulatory blood pressure from baseline to 48 months. (a) Change of the systolic blood pressure. (b) Change of diastolic blood pressure. (c) Change of heart rate. SBP: systolic blood pressure; DBP: diastolic blood pressure; HR, heart rate. ^*∗*^*P* < 0.05, compared with baseline. ^#^*P* < 0.05, GFR46-90 ml/min VS GFR<15 ml/min and *P* < 0.05, GFR46-90 ml/min VS GFR15-45 ml/min.

**Figure 4:**
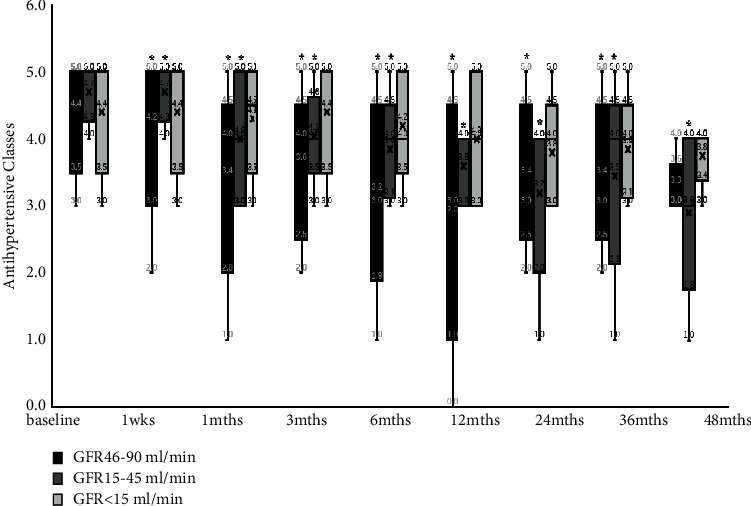
Changes of antihypertensive classes from baseline to 48 months. Note: SBP : systolic blood pressure; DBP : diastolic blood pressure; ^*∗*^*P* < 0.05, compared with baseline.

Baseline SBP was (165.19 ± 6.90)/(91.95 ± 14.47) mm·Hg in the GFR 46–90 ml/min group, (167.63 ± 6.61)/(86.26 ± 10.94) mm·Hg in the 15–45 ml/min group, and (170.14 ± 7.76)/(85.07 ± 10.00) mm·Hg in the GFR<15 ml/min group. There was no difference among the three groups (Figures 3 and 4). Compared with baseline, SBP was significantly decreased in different extents in each group at each follow-up. SBP decreased most in the GFR 46–90 ml/min group (Figure 3(a)).

As shown in Figure 3(b), DBP was decreased lower in the GFR 46–90 ml/min group at each follow-up. But DBP was mildly decreased at some time in the GFR<15 ml/min group such as 1 months of follow-up and in the GFR 15–45 ml/min group such as 6-, 12-, and 24- month follow-up. There was no significant difference in DBP among the three groups.

It can be seen in Figure 3(c) that the changes of the heart rate after RDN treatment. Compared with the baseline, better heart rate control was present to 1 week and 1 month in the GFR 15–45 ml/min group.

**Figure 5:**
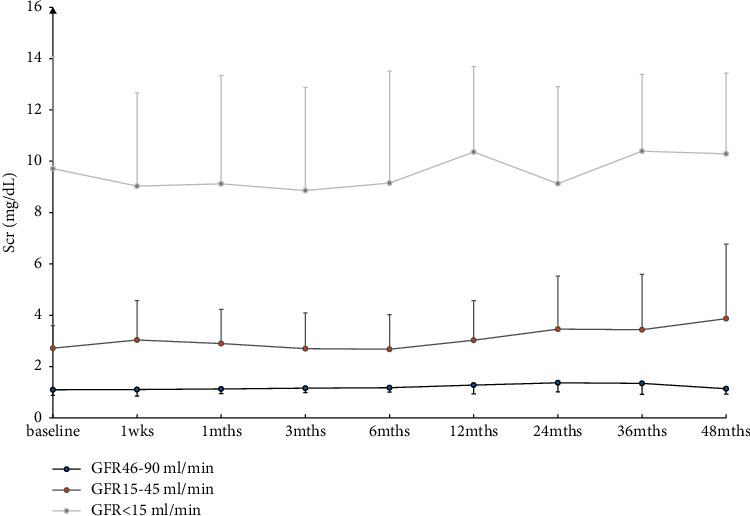
Change of renal function. Note: Scr: serum creatinine and eGFR: estimated glomerular filtration rate hemoglobin (eGFR).

Antihypertensive medication prescription is shown in Figure 4. At baseline, patients were prescribed 4 (4,5) antihypertensive medication classes, which in most patients included an angiotensin receptor blocker or ACE inhibitor, a calcium channel blocker, a diuretic, and a beta-blocker. Compared with baseline, the type of antihypertensive drugs in the GFR46-90 ml/min group decreased significantly except for 36 and 48 months. The type of antihypertensive drugs used in the GFR 15–45 ml/min group decreased significantly at 48 months' postenrolment. No significant reduction in the type of antihypertensive drugs taken in the GFR< 15 ml/min group (Figure 5).

### 3.4. Change of Renal function

The change in eGFR and Scr following RDN is shown in Figure 5. There was no significant difference in Scr and eGFR between the three groups at each follow-up point compared with the baseline.

## 4. Discussion

CKD combined with RHT has always been a worldwide problem with no effective treatment. Traditional treatments have limited efficacy in lowering blood pressure. RHT is often difficult to control blood pressure with antihypertensive drugs. Elevated or fluctuating blood pressure can exacerbate microalbuminuria level, disorder glucose metabolism, and significantly reduce cardiac function [21–23]. Long-term blood pressure fluctuations severely reduce the quality of life of patients and significantly increased the incidence of acute cardiac, cerebral, and vascular events [5]. Previous clinical studies have confirmed the effect on the RDN operation for CKD [24]. However, clinical studies on the efficacy of RDN in patients with different stages of CKD are lacking.

Renal function assessed by eGFR in this cohort of patients with severe, uncontrolled hypertension. In our study, mean body mass index, hemoglobin, and albumin was inversely related to the CKD stage. The incidence of hyperkalemia was high in CKD stage V. These clinical symptoms conventionally appeared in the uremia stage. After RDN, the decrease of systolic blood pressure reached a stable level after 1 month, and the 1-month change in SBP was the largest with CKD patients in GFR15-45 ml/min. SBP remained down to 48 months. The effect was maintained for a long time and complications such as renal artery stenosis, arteriovenous fistula, and death were rare.

This study provided evidence that RDN could lower the blood pressure in patients with CKD complicated with RHT safely and effectively. The sustained reductions in ambulatory SBP are also important because daytime, nighttime, and 24 h ambulatory BP changes are more closely related to cardiovascular risk than office BP measurements. The recently published randomized, sham-controlled SPYRAL HTN-OFF MED [25] SPYRAL HTN-ON MED [26], and RADIANCE SOLO [27] trials documented significant and consistent reductions in both offices and ambulatory BP in patients with and without concomitant antihypertensive medication. Several studies indicated that the trajectory of the progressive decline in the renal function can be altered by RDN.

Secondly, at 48-month postenrollment, patients were prescribed fewer antihypertensive categories, especially in the GFR 15–45 ml/min group. Although changes in prescribed antihypertensive medication regimens were permitted, patients were prescribed slightly clinically meaningful and fewer antihypertensive medication classes at 48 months compared to baseline in the GFR 15–45 ml/min group. Therefore, the reduction in the drug therapy cannot explain the sustained BP decrease after RDN in the patient with GFR 15–45 ml/min. It might reflect a decrease in angiotensin-converting enzyme inhibitors and centrally acting sympatholytic. Previous animal studies and clinical studies have confirmed that hypertensive animal models/patients are characterized by sympathetic hyperactivity, which is the theoretical basis of RDN. During the development of CKD, nephron damage and changes in the renal blood flow could directly or indirectly activate the RAS system and renal sympathetic nervous system. The active substances secreted by the RAS system and renal sympathetic nervous system, such as angiotensin and catecholamine, play a compensatory role in the early stage of CKD, but excessive compensation aggravates the fibrosis and chronic inflammation of the kidney, as well as cardiac and vascular damage, accelerating the development of CKD [28, 29]. Also, the activation of the renal sympathetic nervous system alters central blood pressure regulation by affecting the brain neurons that innervate vasomotion [30].

In addition, this study observed that kinds of antihypertensive drugs increased again at 36 and 48 months in the GFR 46–90 ml/min group. Although medication adherence may have improved in some patients from baseline to follow-up and influenced BP reduction, a recent study [31] showed that nearly 40% of patients were not adherent to their prescribed antihypertensive regimen and that adherence was highly variable among patients at different time points. Meanwhile, patients with end-stage renal disease(GFR<15 ml/min) had lower blood pressure, but no reduction in the type of antihypertensive drugs. With the decrease of the glomerular filtration rate, muscle sympathetic activity increased [32]. More importantly, the density of nerve endings in renal arteries and perineal tissues increased significantly in ESRD patients under the hemodialysis therapy, suggesting that ESRD patients with hemodialysis therapy may have a higher muscle sympathetic nerve activity [33]. In contrast, some studies have shown that the effectively blocking adrenergic receptors and inhibition of sympathetic nerve activity can inhibit the activation of renal apoptotic, inflammatory, and profibrotic factors [34–36]. Several real-world studies have also confirmed that the RDN operation can effectively and safely lower blood pressure in patients with CKD [37]. Therefore, this study is an effective practice and extension of the previous basic pathophysiological research and clinical research. In other words, RDN treatment in CKD stage IIIb might be the best time for intervention and plays a key role in blocking sympathetic excitability and delaying the progression of CKD to end-stage renal disease.

But there were some limitations. Not all patients currently enrolled in ChiCTR-ONC-17012483 study reached 36-month follow-up. At the time of this report, 48 months of follow-up data were available for 50% of the enrolled population. (1) Small-sample, single-center clinical studies can lead to selection bias. (2) We lack effective methods to evaluate the suitability of RDN to population. (3) Comparisons of eGFR measurements between patients with or without medication changes was limited since reported medication changes were not verified with medication adherence testing. (4) The observed safety profile should be regarded as being device-specific. Continued long-term follow-up of patients treated with the newer Symplicity Spyral catheter and revised procedural techniques are required.

## 5. Conclusion

In conclusion, RDN can safely reduce SBP in CKD patients combined with RHT for 48 months, with the most pronounced reduction in the GFR15-45 group. The variety of antihypertensive drugs was significantly decreased after RDN. This was especially evident in patients with GFR 15–45 ml/min. This study expanded the application scope of RDN and provided a new method for the treatment of ESRD combined with RHT, suggesting that RDN interventional treatment in CKD stage CKD IIIb stage might be the best time. In addition, our current study demonstrated a special catheter operation method to improve the success rate of RDN, which is undoubtedly an exciting achievement in the field of RDN and ESRD.

## Figures and Tables

**Table 1:** Subjects and baseline characteristics.

	GFR 46–90 ml/min	GFR 15–45 ml/min	GFR <15 ml/min	*P* value
*N*	21	19	14	
Male (%)	9 (42.85)	7 (36.84)	5 (35.71)	0.89
Hypertension course (y)	3 (3,10)	20 (6,20)	13.75 (10,25)	0.56
Diabetes (%)	10 (47.62)	8 (42.11)	5 (35.71)	0.78
Heart failure (%)	4 (19.05)	5 (26.32)	6 (42.86)	0.30
ASCVD (%)	8 (38.10)	7 (36.84)	5 (35.71)	0.89
Age (y)	51.67 ± 10.73	52.58 ± 13.36	59.07 ± 12.14	0.18
Height (cm)	165.43 ± 9.98	166.53 ± 7.38	164.86 ± 8.76	0.86
Weight (kg)	73.33 ± 20.62	65.95 ± 12.61	62.00 ± 10.95	0.11
BMI (kg/cm^2^)	26.39 ± 4.64	23.34 ± 3.31	22.60 ± 3.17	0.01
HR (bpm)	75.38 ± 13.34	70.26 ± 12.03	71.14 ± 12.17	0.40
Scr (mg/dL)	1.10 ± 0.22	2.72 ± 0.88	9.71 ± 4.37	<0.01
eGFR (ml/min)	79.69 ± 30.18	31.35 ± 9.89	8.59 ± 4.24	<0.01
Hemoglobin (g/L)	133.71 ± 19.21	108.47 ± 18.98	93.14 ± 15.02	<0.01
Thrombocyte (×10^^9^/L)	222.48 ± 61.95	235.37 ± 107.67	205.36 ± 74.20	0.60
HbA1c (%)	6.35 ± 0.88	5.84 ± 0.71	5.38 ± 0.73	<0.01
Total bilirubin (mmol/L)	9.82 ± 4.22	7.86 ± 3.44	5.89 ± 2.80	<0.01
Albumin (g/L)	40.70 ± 6.45	35.99 ± 5.45	34.05 ± 5.46	<0.01
Potassium (mmol/L)	3.92 ± 0.40	4.17 ± 0.68	4.68 ± 0.84	<0.01
Sodium (mmol/L)	141.98 ± 2.97	140.79 ± 2.84	140.89 ± 4.94	0.51
Uric acid (mmol/L)	416.48 ± 125.37	460.61 ± 119.63	441.17 ± 95.54	0.49
Total cholesterol (mmol/L)	4.30 ± 1.16	3.71 ± 0.97	3.71 ± 0.57	0.10
Triglyceride (mmol/L)	2.02 ± 1.05	1.44 ± 0.80	1.06 ± 0.58	<0.01
LDL (mmol/L)	2.18 ± 1.25	1.75 ± 0.65	2.03 ± 0.70	0.35
HDL (mmol/L)	1.31 ± 0.77	1.38 ± 0.83	1.24 ± 0.44	0.87
LAD (cm)	4.27 ± 0.65	4.34 ± 0.41	4.72 ± 0.48	0.04
LVEDd (cm)	5.01 ± 0.62	5.40 ± 0.55	5.74 ± 0.28	<0.01
IVST (cm)	1.26 ± 0.29	1.28 ± 0.22	1.38 ± 0.24	0.39
LVEF (%)	63.95 ± 10.41	58.92 ± 8.91	62.04 ± 5.75	0.21
E/*e*	13.70 ± 5.57	15.05 ± 6.67	17.88 ± 6.88	0.17

Note. Data are mean ± SD, median with interquartile range, or *n* (%). BMI: body mass index; Scr: serum creatinine; eGFR: estimated glomerular filtration rate, male = ((140-age) × kg(/(Scr(mg/dL) ^*∗*^ 72), female = ((140-age) × kg × 0.85)/(Scr(mg/dL) × 72); HbA1c: glycosylated hemoglobin; Scr: serum creatinine; eGFR: estimated glomerular filtration rate hemoglobin(eGFR); LDL: low-density lipoprotein cholesterol; HDL: high-density lipoprotein cholesterol; LAD: left atrial diameter; LVEDd: left ventricular end diastolic diameter; IVST: interventricular septal thickness; LVEF: left ventricular ejection fraction; E/*e*: ratio of early diastolic mitral flow velocity to early diastolic mitral annulus tissue velocity; SBP: systolic blood pressure; DBP: diastolic blood pressure; ASCVD: atherosclerotic cardiovascular disease.

## Data Availability

The majority of the data are held in the Chinese Clinical Trial Registry, under registration number: ChiCTR-ONC-17012483. Referring physician data are available upon request. Data are available from Han Cai and Zhoufei Fang at 64967607@qq.com upon request.
